# Quantification of perforant path fibers for early detection of Alzheimer's disease

**DOI:** 10.1002/alz.70142

**Published:** 2025-04-06

**Authors:** Yuto Uchida, Zhipeng Hou, Laura Gomez‐Isaza, Maria Luongo, Juan C. Troncoso, Michael I. Miller, Susumu Mori, Kenichi Oishi

**Affiliations:** ^1^ Department of Radiology and Radiological Science Johns Hopkins University School of Medicine Baltimore Maryland USA; ^2^ Department of Pathology Division of Neuropathology Johns Hopkins University School of Medicine Baltimore Maryland USA; ^3^ Department of Biomedical Engineering Johns Hopkins University Baltimore Maryland USA; ^4^ Department of Neurology Johns Hopkins University School of Medicine Baltimore Maryland USA; ^5^ The Richman Family Precision Medicine Center of Excellence in Alzheimer's Disease Baltimore Maryland USA

**Keywords:** Alzheimer's disease, diffusion tensor imaging, entorhinal cortex, histology, magnetic resonance imaging, neurodegeneration, perforant path, tractography

## Abstract

**INTRODUCTION:**

The entorhinal cortex (ERC) and perforant path (PP) fibers are critical structures in the pathology of Alzheimer's disease (AD). This study aims to explore these regions using high‐field magnetic resonance imaging (MRI), with the goal of identifying reliable biomarkers based on histopathological observations.

**METHODS:**

Twenty *post mortem* brain specimens were scanned with 11.7T MRI, including diffusion tensor imaging and tractography, and were cut for subsequent histological examinations. The entorhinal cortical thickness and number of PP fibers derived from MRI were compared across neuropathological and *premortem* clinical diagnoses of AD.

**RESULTS:**

The entorhinal cortical thickness and number of PP fibers decreased along with severities of neurofibrillary tangles in the ERC. Meanwhile, a reduction in the number of PP fibers, but not the entorhinal cortical thickness, was observed during the preclinical stage of AD.

**CONCLUSIONS:**

Degeneration of PP fibers was observed in early AD and progressed along with neuropathological changes.

**Highlights:**

- Twenty *post mortem* brain tissues were scanned with 11.7T MRI.
- Degeneration of PP fibers was observed at 250 µm isotropic resolution.
- PP fiber indices were linked with severities of NFTs.
- The number of PP fibers was decreased in preclinical AD.

## 1 BACKGROUND

Early diagnosis of Alzheimer's disease (AD) is crucial for managing symptoms, slowing cognitive decline, and improving patient outcomes.^1^ It enables timely treatment, allows patients and families to make informed decisions about future planning,^2^ and provides access to clinical trials, which can advance research and offer potential medical benefits.^3^ Structural brain magnetic resonance imaging (MRI) is a valuable tool in detecting atrophy patterns characteristic of AD‐related neurodegeneration, which correlate well with disease stage and pathology.^4^ For instance, measuring the cortical thickness in the entorhinal cortex (ERC) may serve as a potential neurodegenerative biomarker for AD.^5^, ^6^, ^7^ However, the sensitivity and specificity of current structural MRI‐based morphological measures in detecting the earliest stages of AD may be relatively low compared to other biomarkers.^8^, ^9^, ^10^ Identifying anatomical features that are better related to AD‐specific pathology than current morphological measures is highly anticipated to overcome current limitations.^11^

Degeneration of perforant path (PP) fibers is one of the candidates for the highly sensitive and specific biomarkers of the AD continuum.^12^ Histopathological studies using autopsy samples have shown that the PP is the first site where neurofibrillary tangles (NFTs), composed of hyperphosphorylated tau, emerge during the early stages without AD symptoms, that is, preclinical stage of AD.^13^, ^14^, ^15^ Anatomically, the PP represents the major route of input to the hippocampus, with axons that originate within the ERC and project to the dentate gyrus and Ammon's horn through the presubiculum.^16^ This tract functions as an essential node in a larger network, transmitting information from various neocortical association areas through the ERC and hippocampus and eventually disseminating it throughout the limbic system.^17^, ^18^ Degeneration of PP fibers is related to memory deterioration because it results in the functional disconnection of higher‐level sensory input to the hippocampus.^19^, ^20^ These pathological and functional backgrounds of the PP make its MRI finding a potential imaging biomarker for neurodegeneration during the preclinical stage of AD.

A few studies tackled challenges in quantifying PP fibers using diffusion tensor imaging (DTI) and tractography.^21^, ^22^ Since the average width of the entorhinal layer II islands, where PP fibers originate, is approximately 500 µm,^23^ image acquisition at a submillimeter resolution is required. To accurately quantify the parameters of PP fibers, including their numbers, length, and fractional anisotropy (FA) values, and compare the findings with histopathological evaluations, an ex vivo MRI study at the ultrahigh‐field strength is essential.^24^ In our previous study of *post mortem* human brain tissues scanned with 11.7T MRI, followed by histological verification, we observed the degeneration of PP fibers, indicated by lower fiber counts, reduced FA values, and diminished T2‐weighted signal contrasts.^25^ Furthermore, histological analyses demonstrated that areas of PP fibers on the presubiculum decreased along the AD continuum.^25^ However, it remains an open question whether these changes can be seen by MRI during the preclinical stage of AD and related to severities of AD neuropathological changes.

Given the pathological and functional significance of the PP to the earliest AD pathogenesis, we hypothesized that its myeloarchitectonic features detected using 11.7T MRI ex vivo would be associated with severities of AD pathological changes, providing a basis for further research into non‐invasive biomarkers for neurodegeneration in the process of AD pathogenesis. To test this hypothesis, we used *post mortem* human brain tissues from non‐AD, preclinical AD, and AD dementia to investigate whether the T2‐weighted contrast, DTI, and tractography scanned with 11.7T MRI ex vivo were related to the histologically confirmed degeneration of PP fibers.

## 2 METHODS

### 2.1 Human brain specimens

This study was performed under a protocol for the use of de‐identified human brain tissues for research purposes, approved by the Institutional Review Board of Johns Hopkins University School of Medicine. The age was masked if it was 90 years or older due to concerns about potential identification of individuals. A total of 20 *post mortem* brain specimens of the left cerebral hemisphere were provided by the Brain Resource Center at the Johns Hopkins Alzheimer's Disease Research Center. Clinical assessments of cognition were made based on the *premortem* clinical dementia rating (CDR) scales. Neuropathological assessments^26^ and clinical staging of AD^27^ were made based on the National Institute on Aging and Alzheimer's Association (NIA‐AA) guidelines.

**RESEARCH IN CONTEXT**

**Systematic review**: We reviewed PubMed literature on associations between the PP and AD neuropathological changes. While the degeneration in PP fibers has been shown along with AD neuropathological changes, it remains to be elucidated whether its neurodegeneration can be detected during the preclinical stage of AD.

**Interpretation**: Using ex vivo 11.7T diffusion MRI at 250 µm isotropic resolution, we demonstrated the alterations of fractional anisotropy on the subiculum and the number of tractograms of PP fibers during the preclinical stage of AD. These findings highlight the potential of microstructural neurodegeneration detected by diffusion MRI at ultrahigh‐field strength as an emerging biomarker to monitor disease progression along the AD continuum.

**Future directions**: Moving forward, it will be interesting to explore how this proof‐of‐concept research can be translated to lower‐resolution MRI scans in vivo, considering variations in signal‐to‐noise ratios, shorter scan times, and potential motion artifacts.

### 2.2 Histological observation

The brain specimens were fixed in 10% formaldehyde over 2 weeks and sectioned into 10‐mm‐thick coronal slabs. Tissue blocks, approximately 30 × 20 mm in size, were cut from the slabs extending from the temporal pole to the hippocampal tail. After MRI scans, the brain tissues were embedded in paraffin blocks, cut into 10‐µm‐thick sections at 200‐µm intervals, and stained using Luxol fast blue with hematoxylin and eosin (LFB&HE) for histological examination of myelinated fibers and neuronal cells. For immunohistochemistry to obtain an “ABC score” of AD neuropathological changes,^26^ the 6E10 (1:500, BioLegend San Diego, CA, USA) and AT8 antibodies (MN1020, Thermo Fisher Scientific, Waltham, MA, USA) were used to label the amyloid beta (Aβ) and phosphorylated tau pathologies, respectively. Briefly, the ABC score combines the Thal amyloid phase (A), Braak NFT stage (B), and Consortium to Establish a Registry for AD (CERAD) score (C), resulting in a composite score of 0 to 3 in ABC scores, respectively (Table S1). Alpha‐synuclein and TAR DNA‐binding protein 43 (TDP‐43) were also immunostained and evaluated using the neuropathological consensus criteria for Lewy pathology^28^ and TDP‐43 proteinopathy,^29^ respectively. Histopathological images were captured using a Zeiss Axio Observer.Z1 microscope equipped with an AxioCam MRc camera (Carl Zeiss Microscopy, Thornwood, NY, USA) and a ×5 objective lens. NFTs in the ERC were counted to ascertain the associations with the MRI findings and thereby semi‐quantified as follows: 0 = none; 1 to 3 = sparse; 4 to 9 = moderate; >10 = frequent.

### 2.3 MRI scan and processing

Ultrahigh‐field anatomical MRI was acquired using an 11.7T NMR spectrometer (Bruker Biospin, Billerica, MA, USA). A single‐channel 30‐mm Bruker volume coil was used for both radio frequency transmission and reception. For MRI scans, the brain tissues were transferred to PBS with 2 mM gadopentetate dimeglumine for 48 h. They were then placed inside 50‐mL conical tubes filled with proton‐free liquid (Fomblin: Ausimont, Thorofare, NJ, USA). Air bubbles were removed by placing the samples in a vacuum chamber for more than 30 min before taking the MRI scans. To acquire three‐dimensional T2‐weighted images (3DT2WI) using rapid acquisition with relaxation enhancement (RARE), the following parameters were applied: echo time = 18 ms; repetition time = 2500 ms; RARE factor = 8; signal average = 4. The field of view was 40 × 30 × 16 mm^3^, and the matrix size was 160 × 120 × 64, which was zero‐filled to 320 × 240 × 128, resulting in a final resolution of 250 µm isotropic (zipped to 125 µm isotropic). For DTI and tractography, diffusion‐weighted gradient and spin echo sequences with navigator phase correction were applied to the ex vivo brain tissue.^30^ The scan parameters were as follows: echo time = 58 ms; repetition time = 600 ms; 2 signal averages; 2 b0 images; and 18 diffusion directions with *b*‐value = 2000 s/mm^2^. The temperature during the scan was 27°C. The field of view and matrix size were the same as those of 3DT2WI. The total scan time for each brain tissue was 10 h.

DtiStudio software (https://www.MRIstudio.org)^31^ was used for tensor calculation. The linear registration method minimized a cost function based on mean square tensor fitting errors to correct eddy current distortion and motion of the tissue.^32^ The pixels with artifactual signal were eliminated from the tensor calculation using the corrected Inter‐Slice Intensity Discontinuity algorithm.^33^ Three eigenvalues were extracted from the tensor field to calculate FA values. The FA map was color‐coded by the principal eigenvectors shown in red (medial–lateral orientation), green (anterior–posterior orientation), and blue (superior–inferior orientation). The Gibbs ringing artifact^34^ was removed from all the images using MRtrix3 software (https://mrtrix.org; RRID:SCR_006971).^35^

### 2.4 Microstructural analysis

**FIGURE 1:**
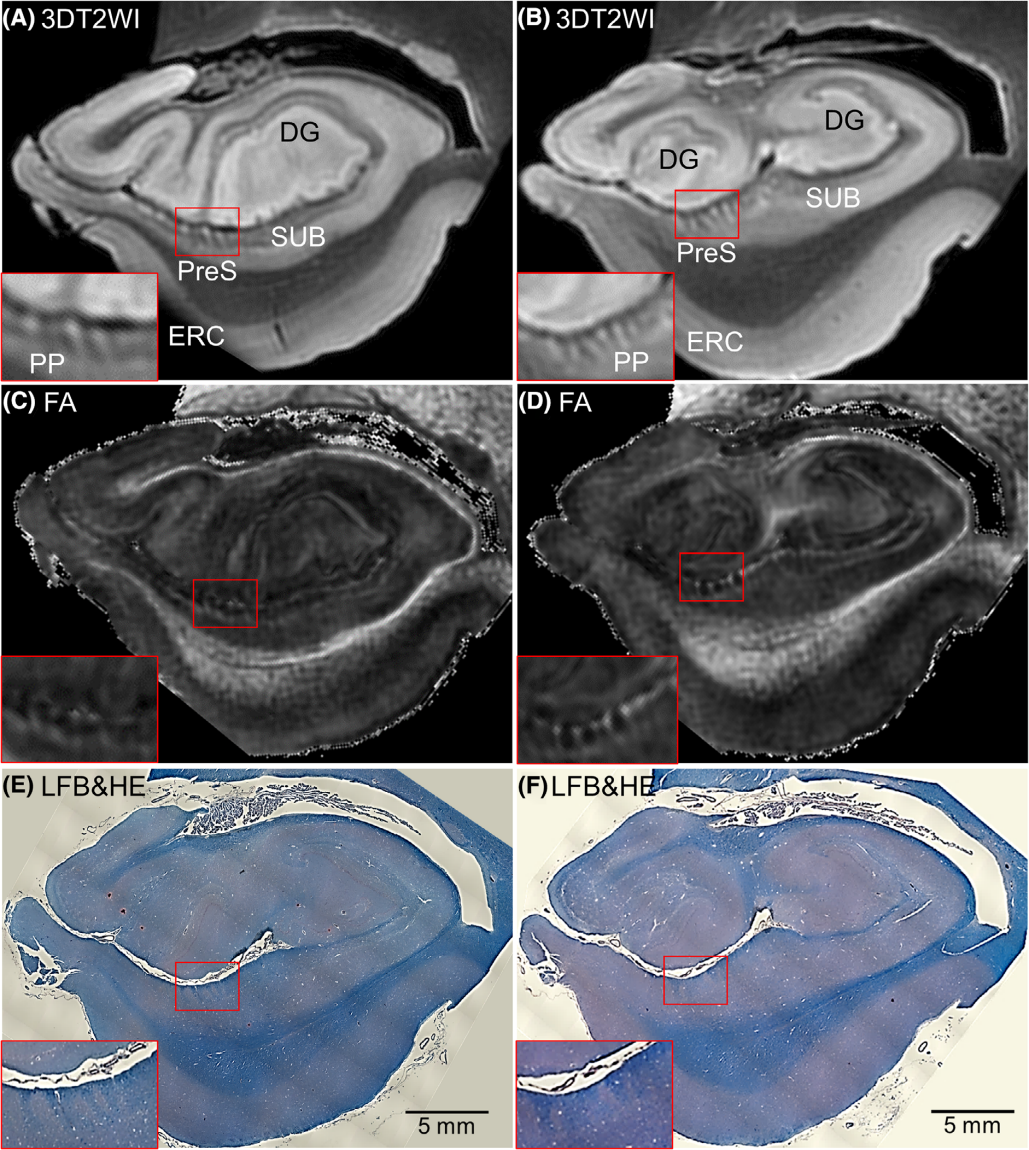
11.7T MRI coronal panels of left side of medial temporal lobe in representative non‐Alzheimer's disease (AD) brain tissue. The red bounding boxes are 5× magnified to clearly visualize the perforant pathway (PP) on the presubiculum (PreS). (A and B) Three‐dimensional T2‐weighted images (3DT2WI). PP, indicated as dark striate intensities, is a set of fibers projecting from the entorhinal cortex (ERC) through PreS to the dentate gyrus (DG) in the hippocampus. DG of the hippocampal head is prominently observed in the slice shown in (A). In the slice shown in (B), which is positioned slightly more caudally, the DG of the hippocampal head is split into inner and outer portions. (C and D) Fractional anisotropy (FA) map. The PP on PreS appeared as bright striates. (E and F) Luxol fast blue with hematoxylin and eosin staining (LFB&HE). Blue areas: PP on PreS. SUB, subiculum.

At 11.7T MRI, the PP was discernible within the presubiculum, which was validated by subsequent serial histological examinations, as described previously.^25^ At an isotropic resolution of 250 µm (zipped to 125 µm isotropic resolution), the voxels occupied by PP fibers showed dark striate intensities on the presubiculum in 3DT2WI (Figure 1A,B and Figure S1A). We counted the number of these voxels and measured the ratio to the overall area of the presubiculum using the RoiEditor software (https://www.MRIstudio.org) on the following slices of 2D coronal planes in the left medial temporal lobe. The criteria used to select the coronal slices were from the first slice, where the dentate gyrus of the hippocampus was initially observed (Figure 1A), to the last slice, where the dentate gyrus of the hippocampus was split into the inner and outer portions (Figure 1B). Herein, we named this ratio the PP ratio (Figure S1B). In the same coronal slices of the FA map as those of 3DT2WI, the mean FA value of the presubiculum was calculated using RoiEditor software. Moreover, in the corresponding coronal slices of 3DT2WI, the thickness of the ERC was measured using ITK‐SNAP software (http://www.itksnap.org; Figure S2).

### 2.5 Tract reconstruction

To output the number and length of PP fibers, the fiber assignment by continuous tracking (FACT) deterministic algorithm^36^ was used for the reconstruction of PP fibers, which is implemented in DtiStudio. A FA threshold of 0.1, an angle threshold of 60°, and a minimum length of five pixels were applied to determine the fiber tract.^37^ An “OR” operation was set on the presubiculum for the first seed point where myelinated PP fibers were seen on the FA map. The FA map was primarily used as a reference to identify the seed point since its image contrast was comparable to that of the myelin‐stained histological section.^38^ The corresponding color‐coded FA map was also used to guide the placement of the seed points. Then an “AND” operation was set on the angular bundle for the second seed point. Following these steps, the PP was automatically reconstructed as a tract connecting the ERC and the dentate gyrus of the hippocampus through the presubiculum (Video S1). Anatomically implausible fibers for a portion of PP fibers were removed using a “NOT” operation as an additional step.

### 2.6 Statistics

Differences in anatomical MRI parameters among the pathologically and clinically diagnosed brain tissues were initially evaluated using an analysis of covariance (ANCOVA) with the adjustment for potential confounders, including age, sex, and brain weight. The assumptions of linearity, homoscedasticity, and normality of residuals were tested prior to conducting ANCOVA, and no violations were detected. For significant findings from the initial group comparisons, pairwise post hoc *t*‐tests were performed using the adjusted means derived from ANCOVA. The *p* values obtained from these pairwise *t*‐tests were subsequently corrected using the Benjamini–Hochberg procedure to control the false discovery rate (FDR) for multiple comparisons. Specifically, this correction was applied to 5 × 6 comparisons across five MRI parameters in six pairwise group comparisons of AD neuropathological changes and 5 × 3 comparisons across the same five MRI parameters in three pairwise group comparisons of clinical AD diagnoses. Significant differences are denoted as *FDR‐corrected *p* value < .05 and **FDR‐corrected *p* value < .01.

For participants aged ≥90 years, we only received information indicating that they were over 90 years old without access to their exact ages, following the guidelines provided by the local ethics committee. In the main analysis, these participants were treated as 90 years old. To ensure that this assumption did not introduce significant bias, a sensitivity analysis was performed by varying the assumed ages (90, 95, and 100 years old). Additionally, to evaluate the robustness of the tractography‐derived measurements, a sensitivity analysis was also conducted by varying the tractography parameters. Specifically, the FA threshold (0.05, 0.10, 0.15), angle threshold (50°, 60°, and 70°), and minimum fiber length (4, 5, and 6 pixels) were adjusted to assess their effects on PP fiber counts and lengths.

## 3 RESULTS

### 3.1 Human brain tissues

Demographics and pathological findings of human brain tissues are summarized in Table 1. Among 20 brain tissues, two experimental groups were analyzed: 10 AD cases, all pathologically diagnosed with low, intermediate, or high AD neuropathological changes and clinically classified as preclinical AD or AD dementia based on the CDR scales; and 10 non‐AD cases. Age at the time of death ranged from 48 to over 90 years; eight participants were female and 12 male; 19 were White, and one was Black. In all AD cases, Braak NFT stages were III or higher and had moderate/frequent NFTs in the ERC. One non‐AD case was co‐diagnosed with limbic‐predominant age‐related TDP‐43 encephalopathy (LATE), characterized by TDP‐43 immunoreactive beaded dystrophic neurites in the hippocampus, and no cases exhibited Lewy body pathology. There were no significant differences in age, sex, or brain weight across the neuropathological (Table S2) and clinical diagnoses of AD (Table S3).

**TABLE 1:** Demographics, neuropathological, and clinical features of individuals.

Case	Age, y	Sex	CDR	Brain weight (g)	NIA‐AA Dx for AD neuropathologic change (ABC score)	Braak NFT stage	Lewy body	TDP‐43	NFTs in entorhinal cortex	NIA‐AA Dx for clinical cognitive staging combined with biomarkers
1	48	Male	0	1440	Not (A0, B1, C0)	II	Negative	Negative	Sparse	Non‐AD
2	50	Male	0	1590	Not (A0, B0, C0)	0	Negative	Negative	None	Non‐AD
3	53	Female	0	1330	Not (A0, B0, C0)	0	Negative	Negative	None	Non‐AD
4	55	Female	0	1250	Not (A0, B1, C0)	II	Negative	Negative	Moderate	Non‐AD
5	57	Male	0	1610	Not (A0, B1, C0)	III	Negative	Negative	Sparse	Non‐AD
6	59	Male	0	1510	Not (A0, B0, C0)	0	Negative	Negative	None	Non‐AD
7	65	Male	0	1470	Not (A0, B1, C0)	I	Negative	Negative	Sparse	Non‐AD
8	80	Male	0	1240	Not (A0, B1, C0)	I	Negative	Negative	Sparse	Non‐AD
9	>90	Female	0	1070	Not (A0, B1, C0)	II	Negative	Negative	Sparse	Non‐AD
10	71	Male	1	1430	Not (A0, B2, C0)	III	Negative	Positive	Frequent	Non‐AD, LATE
11	57	Male	0	1190	Low (A1, B2, C1)	IV	Negative	Negative	Moderate	Preclinical AD
12	67	Male	0	1330	Intermediate (A2, B2, C2)	IV	Negative	Negative	Moderate	Preclinical AD
13	68	Female	0	1210	Intermediate (A2, B2, C2)	IV	Negative	Negative	Moderate	Preclinical AD
14	78	Female	0	1120	Low (A1, B2, C1)	III	Negative	Negative	Moderate	Preclinical AD
15	90	Male	0	1410	Intermediate (A1, B3, C3)	III	Negative	Negative	Frequent	Preclinical AD
16	>90	Female	0	1220	Low (A1, B2, C1)	III	Negative	Negative	Moderate	Preclinical AD
17	75	Female	2	1450	Intermediate (A2, B2, C2)	IV	Negative	Negative	Frequent	AD dementia
18	83	Male	2	1400	High (A3, B3, C3)	VI	Negative	Negative	Moderate	AD dementia
19	>90	Male	3	1290	Intermediate (A3, B2, C2)	IV	Negative	Negative	Frequent	AD dementia
20	>90	Female	3	1180	High (A3, B3, C3)	VI	Negative	Negative	Frequent	AD dementia

*Note*: Negative indicates that no pathology was observed on any examined slide, while positive indicates the presence of pathology based on Lewy^28^ and TDP‐43^29^ neuropathological criteria.

Abbreviations: AD, Alzheimer's disease; CDR, Clinical Dementia Rating; Dx, diagnosis; LATE, limbic‐predominant age‐related TDP‐43 encephalopathy; NFT, neurofibrillary tangle; NIA‐AA, National Institute on Aging and Alzheimer's Association; TDP‐43, TAR DNA‐binding protein 43.

### 3.2 Identification of anatomical structures

**FIGURE 2:**
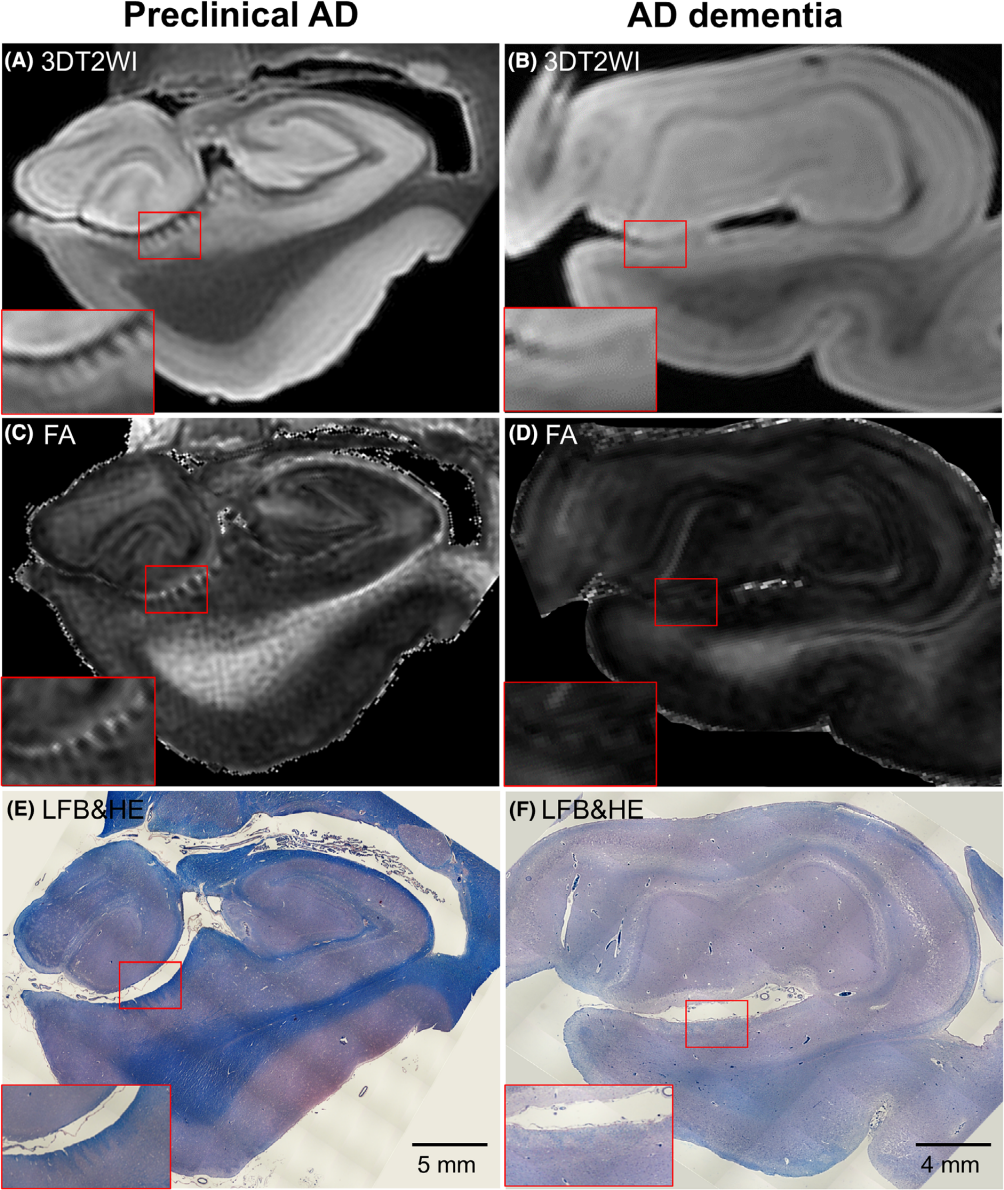
11.7T MRI coronal panels of left entorhinal cortex in representative preclinical Alzheimer's disease (AD) and AD dementia brain tissues. (A and B) Three‐dimensional T2‐weighted images (3DT2WI). (C and D) Fractional anisotropy (FA) map. (E and F) Luxol fast blue with hematoxylin and eosin staining (LFB&HE). The red bounding boxes are 5× magnified to clearly visualize the perforant pathway (PP) on the presubiculum. Note that the PP fibers are not discernible in the AD dementia brain tissues.

At visual inspection, dark striate intensities of PP fibers on the presubiculum in non‐AD (Figure 1A,B) and preclinical AD brain tissues (Figure 2A) were seen in 3DT2WI. In addition, bright striates of PP fibers on the presubiculum in non‐AD (Figure 1C,D) and preclinical AD brain tissues (Figure 2C) were seen in FA maps. In contrast, these maps in AD dementia brain tissues displayed fewer contrasts, which revealed the demise of PP fibers (Figure 2B,D). On microscopic observations, PP fibers showed blue contrasts in LFB&HE, which were clearly visible in non‐AD (Figure 1E,F) and preclinical AD (Figure 2E), whereas they were indiscernible in AD dementia brain tissues (Figure 2F).

### 3.3 Quantitative MRI analyses across AD neuropathological changes

**FIGURE 3:**
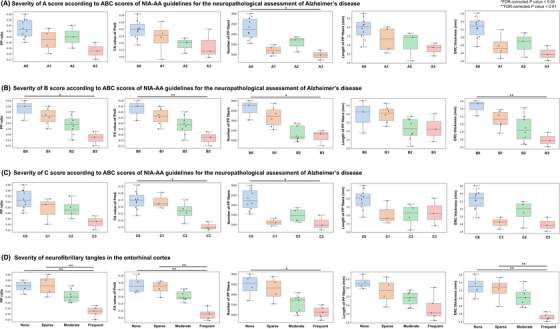
Anatomical MRI parameters across the groups of AD neuropathological changes. The perforant path (PP) ratio, mean fractional anisotropy (FA) value of the presubiculum (PreS), number and length of PP fibers, and entorhinal cortex (ERC) thickness were compared across severities of ABC (A: Thal amyloid phase; B: Braak NFT stage; C: CERAD score) scores of AD neuropathological changes (A–C) and NFTs in ERC (D). The PP ratio, mean FA value of PreS, number of PP fibers, and ERC thickness decreased along severities of B scores of AD neuropathological changes and NFTs in ERC. The differences in these parameters were more prominent due to the degree of severities in NFTs than that of amyloid plaques. While the length of PP fibers did not differ significantly among the groups, the number of PP fibers showed significant differences.

Anatomical MRI parameters of PP fibers, including the PP ratio in 3DT2WI, the mean FA value of the presubiculum, and the number and length of PP fibers derived from DTI tractography, and the ERC thickness were compared across severities of ABC scores in AD neuropathological changes and NFTs in the ERC (Figure 3). The PP ratio, mean FA value of the presubiculum, number of PP fibers, and ERC thickness decreased along with severities of B scores and those of NFTs in the ERC. Notably, the differences in these parameters were more prominent due to the degree of severities in NFTs than that of amyloid plaques. While the length of PP fibers did not differ significantly among the groups, the number of PP fibers showed significant differences across groups.

### 3.4 Quantitative MRI analyses across clinical AD diagnoses

**FIGURE 4:**
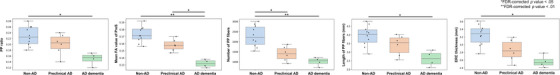
Anatomical MRI parameters across the groups of clinical AD diagnoses. The perforant path (PP) ratio, mean fractional anisotropy (FA) value of the presubiculum (PreS), number and length of PP fibers, and entorhinal cortex (ERC) thickness were compared across the groups along the AD continuum (non‐AD vs preclinical AD vs AD dementia). All MRI parameters in AD dementia were lower than those in non‐AD brain tissues. Additionally, there were significant differences in the mean FA value of PreS between the preclinical AD and AD dementia brain tissues (mean difference = 0.038, 95% confidence interval [CI] = 0.014 to 0.059, false discovery rate (FDR)‐corrected *p* = 0.005) and the number of PP fibers between the non‐AD and preclinical AD brain tissues (mean difference = 759, 95% CI = 289 to 1225, FDR‐corrected *p* = 0.019). In contrast to the MRI parameters derived from PP fibers, a significant difference in ERC thickness was observed only between the non‐AD and AD dementia brain tissues (mean difference = 0.767, 95% CI = 0.145 to 1.289, FDR‐corrected *p* = 0.012).

The MRI parameters that were applied to the quantitative analyses in AD neuropathological changes were compared among the clinical AD diagnoses along with the AD continuum (non‐AD vs preclinical AD vs AD dementia; Figure 4). The PP ratio, mean FA value of the presubiculum, number and length of PP fibers, and ERC thickness in AD dementia were lower than those in non‐AD brain tissues. Additionally, there were significant differences in the mean FA value of the presubiculum between the preclinical AD and AD dementia brain tissues (mean difference = 0.038, 95% CI = 0.014 to 0.059, FDR‐corrected *p* = 0.005) and the number of PP fibers between the non‐AD and preclinical AD brain tissues (mean difference = 759, 95% CI = 289 to 1225, FDR‐corrected *p* = 0.019). In contrast to the MRI parameters derived from PP fibers, a significant difference in the thickness of the ERC was observed only between the non‐AD and AD dementia brain tissues (mean difference = 0.767, 95% CI = 0.145 to 1.289, FDR‐corrected *p* = 0.012).

The sensitivity analysis, performed with variations in the assumed ages and with the exclusion of the non‐AD case with LATE, indicated that these changes did not affect the statistical significance of group differences (Table S4). Similarly, the sensitivity analysis, conducted with variations in the tractography parameters, demonstrated that changes in fiber counts and lengths remained within ± 10% of the baseline values across all parameter variations (Table S5).

## 4 DISCUSSION

In this study, we acquired 20 *post mortem* human brain specimens after confirming the neuropathological assessments and *premortem* clinical diagnoses of AD according to the NIA‐AA guidelines. The medial temporal lobes of these brain tissues were scanned using 11.7T MRI ex vivo for 3DT2WI, DTI, and tractography in submillimeter resolutions. Then we quantified the MRI parameters, such as the PP ratio, FA value of the presubiculum, number and length of PP fibers, and thickness of the ERC, and compared these quantitative values with neuropathological findings related to AD pathogenesis, including the degree of accumulation of amyloid plaques and NFTs in the medial temporal lobes. The primary finding was that greater degeneration of PP fibers, detectable via 11.7T MRI ex vivo, was associated with histologically confirmed higher severity of NFTs in the ERC. Furthermore, we detected a significant reduction in the number of PP fibers but not ERC thickness during the preclinical stage of AD in this small dataset. While numerous MRI studies have focused on the thinning of the ERC that preceded clinical symptoms,^6^, ^39^, ^40^, ^41^ the findings of the present study supported the hypothesis that degeneration of PP fibers, as assessed by ultrahigh‐field MRI parameters, would be more pronounced across the AD continuum (non‐AD < preclinical AD < AD dementia).

Using DTI and tractography at an isotropic resolution of 250 µm (zipped to 125 µm isotropic resolution), we measured the FA value of the presubiculum in the FA map and the number and length of PP fibers derived from streamlines created by the FACT algorithm.^36^ The reduced FA value and decreased number of PP fibers are thought to result from demyelination, axonal degeneration, or both during AD pathogenesis.^42^, ^43^ It is important to note that diffusion MRI does not directly visualize axons but only provides indirect information based on diffusions of surrounding water molecules. To address this biological non‐specificity, we first demonstrated that the areas of myelinated fibers identified by histological analysis corresponded to the areas visualized by streamlines from tractography. The reconstructed trajectories of the PP in this study aligned with those previously observed using polarized light microscopy in the human hippocampus ex vivo,^44^ validating our tractography method. Moreover, while the length of PP fibers remained preserved in preclinical AD, the number of PP fibers was significantly decreased from the preclinical stage of AD, consistent with previous studies of DTI and tractography.^25^, ^43^

The MRI parameters we selected in this study for quantifying PP fibers were derived not only from quantitative MRI techniques, such as DTI and tractography, but also measured using 3DT2WI. The T2‐weighted signal with ultrahigh‐field strength and high‐performance gradients can provide good contrasts between soft tissues, making it useful for visualizing various brain structures and pathologies.^45^ Low signal contrasts in neuronal fibers on 3DT2WI can be generated due to the following reasons: (1) myelinated nerve fibers tend to have low signal intensity on 3DT2WI because the myelin content produces a shorter T2 relaxation time, resulting in darker contrasts^46^; (2) high‐density fiber tracts can also appear darker due to less water content and more tightly packed myelin^47^; (3) certain pathological conditions, including chronic demyelination or gliosis, might alter the signal characteristics, making the neuronal fibers appear brighter compared to non‐pathological status.^48^ Low T2‐weighted signal contrasts on the presubiculum, consistent with PP fibers, confirmed by histological verification, led us to define the PP ratio and demonstrate its association with neuropathological findings related to AD pathogenesis.

In this study, DTI and tractography directly extracted quantitative measures, including the FA value of the presubiculum and the number and length of PP fibers. A previous work by Granger et al.^49^ also highlighted the potential of using high‐resolution diffusion MRI to detect PP degeneration and its relationship to memory impairment. However, these strategies have limitations for clinical application due to the longer scan time and lower spatial resolutions.^50^ On the other hand, 3DT2WI is a conventional imaging method widely used in clinical practice, enabling us to acquire images with higher spatial resolutions and signal‐to‐noise ratios.^45^ The utilization of 3DT2WI is expected to be necessary for visualizing PP fibers in vivo in clinical application as a neuroimaging biomarker for the neurodegeneration of AD.^25^ Radiomics‐based approaches, particularly those involving hippocampal texture analysis, have shown promise in detecting microstructural changes in early AD.^51^

This study has several limitations. First, caution should be exercised when interpreting our results as an AD‐specific neurodegeneration. Considering the age‐dependent degradation of PP fibers,^22^ the findings might reflect both age‐ and non‐AD‐related neurodegeneration. To account for the effects of aging, we included the demographics, including age, sex, and brain weight, as covariates in the statistical analyses. Regarding the co‐pathological changes other than AD, there were no cases with Lewy body pathological changes and only one case with TDP‐43 immunoreactive beaded dystrophic neurites in the hippocampus. A longitudinal study for cognitively unimpaired individuals with and without abnormal AD biomarkers is necessary for investigating whether the MRI findings of PP fibers can be used as biomarkers for neurodegeneration of AD pathogenesis. Second, we did not obtain memory function test scores in this cohort, resulting in the inability to analyze the associations between cognitive function scores and quantitative parameters derived from the MRI findings of PP fibers. In previous studies of diffusion MRI, the lower the FA value of PP fibers, the lower the episodic memory function test was before atrophic changes of the medial temporal lobe.^22^, ^52^ Third, the preprocessing steps of ex vivo histological examinations would affect the quantitative MRI measurements, such as the *post mortem* interval and fixation, though prior studies indicated that FA values remained stable up to 72 h *post mortem* ^53^ and that fixation does not significantly alter FA values.^54^ Finally, the racial and ethnic homogeneity of our sample may limit the generalizability of our findings. Given that racial and ethnic differences have been reported in AD pathology,^55^ future studies should incorporate more diverse cohorts to enhance the broader applicability of our results.

In conclusion, this study demonstrated that ultrahigh‐field 11.7T MRI ex vivo could detect microstructural degeneration of PP fibers. Quantitative MRI parameters derived from 3DT2WI, DTI, and tractography at submillimeter resolutions differed among the severity of AD neuropathological changes. Subsequent serial histological examinations validated the MRI findings reflective of the myeloarchitectonic features of PP fibers. In the preclinical stage of AD, the number of PP fibers was detectable, whereas the thickness of the ERC was not. These results pave the way for the development of highly sensitive biomarkers for neurodegeneration, enabling the detection of microstructural changes in myelinated fibers during the early stages of AD pathogenesis. Moving forward, it will be interesting to explore how this proof‐of‐concept research can be translated to lower‐resolution MRI scans in vivo, considering variations in signal‐to‐noise ratios, shorter scan times, and potential motion artifacts.

## CONFLICT OF INTEREST STATEMENT

M.I.M. and S.M. are co‐founders of and hold equity stakes in AnatomyWorks. K.O. is a consultant to Anatomy Works. Under a license agreement between AnatomyWorks and Johns Hopkins University, M.I.M., S.M., K.O., and the University are entitled to royalty distributions related to technology described in the study. S.M. is the founder and CEO of Corporate M. This arrangement has been reviewed and approved by the Johns Hopkins University in accordance with its conflict‐of‐interest policies. Y.U., Z.H., L.G.I., M.L., and J.C.T. declare no conflicts of interest. Author disclosures are available in the supporting information.

## CONSENT STATEMENT

This study was performed under a protocol for the use of de‐identified human brain tissues for research purposes, approved by the Institutional Review Board of Johns Hopkins University School of Medicine, confirming that consent was not necessary.

## Supporting information



Supporting Information

Supporting Information

Supporting Information

## References

[1] Livingston G , Huntley J , Liu KY , et al. Dementia prevention, intervention, and care: 2024 report of the Lancet standing Commission. Lancet. 2024;404:572‐628. 39096926 10.1016/S0140-6736(24)01296-0

[2] Rasmussen J , Langerman H . Alzheimer's disease—why we need early diagnosis. Degener Neurol Neuromuscul Dis. 2019;9:123‐130. 31920420 10.2147/DNND.S228939 PMC6935598

[3] Zhang J , Zhang Y , Wang J , Xia Y , Zhang J , Chen L . Recent advances in Alzheimer's disease: mechanisms, clinical trials and new drug development strategies. Signal Transduct Target Ther. 2024;9:211. 39174535 10.1038/s41392-024-01911-3 PMC11344989

[4] McEvoy LK , Brewer JB . Quantitative structural MRI for early detection of Alzheimer's disease. Expert Rev Neurother. 2010;10:1675‐1688. 20977326 10.1586/ern.10.162 PMC3182103

[5] Holbrook AJ , Tustison NJ , Marquez F , et al. Anterolateral entorhinal cortex thickness as a new biomarker for early detection of Alzheimer's disease. Alzheimers Dement. 2020;12:e12068. 10.1002/dad2.12068 PMC7447874 32875052

[6] Kulason S , Xu E , Tward DJ , et al. Entorhinal and transentorhinal atrophy in preclinical Alzheimer's disease. Front Neurosci. 2020;14:804. 32973425 10.3389/fnins.2020.00804 PMC7472871

[7] Thaker AA , Weinberg BD , Dillon WP , et al. Entorhinal cortex: antemortem cortical thickness and postmortem neurofibrillary tangles and amyloid pathology. AJNR Am J Neuroradiol. 2017;38:961‐965. 28279988 10.3174/ajnr.A5133 PMC5433913

[8] Lombardi G , Crescioli G , Cavedo E , et al. Structural magnetic resonance imaging for the early diagnosis of dementia due to Alzheimer's disease in people with mild cognitive impairment. Cochrane Database Syst Rev. 2020;3:Cd009628. 32119112 10.1002/14651858.CD009628.pub2 PMC7059964

[9] Márquez F , Yassa MA . Neuroimaging biomarkers for Alzheimer's disease. Mol Neurodegener. 2019;14:21. 31174557 10.1186/s13024-019-0325-5 PMC6555939

[10] Dang C , Wang Y , Li Q , Lu Y . Neuroimaging modalities in the detection of Alzheimer's disease‐associated biomarkers. Psychoradiology. 2023;3:kkad009. 38666112 10.1093/psyrad/kkad009 PMC11003434

[11] Sabbagh MN , Boada M , Borson S , et al. Rationale for early diagnosis of mild cognitive impairment (MCI) supported by emerging digital technologies. J Prev Alzheimers Dis. 2020;7:158‐164. 32463068 10.14283/jpad.2020.19

[12] García‐Sierra F , Hauw JJ , Duyckaerts C , Wischik CM , Luna‐Muñoz J , Mena R . The extent of neurofibrillary pathology in perforant pathway neurons is the key determinant of dementia in the very old. Acta Neuropathol. 2000;100:29‐35. 10912917 10.1007/s004010051189

[13] Hyman BT , Van Hoesen GW , Damasio AR , Barnes CL . Alzheimer's disease: cell‐specific pathology isolates the hippocampal formation. Science. 1984;225:1168‐1170. 6474172 10.1126/science.6474172

[14] Braak H , Braak E . On areas of transition between entorhinal allocortex and temporal isocortex in the human brain. Normal morphology and lamina‐specific pathology in Alzheimer's disease. Acta Neuropathol. 1985;68:325‐332. 4090943 10.1007/BF00690836

[15] Braak H , Braak E . Neuropathological stageing of Alzheimer‐related changes. Acta Neuropathol. 1991;82:239‐259. 1759558 10.1007/BF00308809

[16] Witter MP . The perforant path: projections from the entorhinal cortex to the dentate gyrus. Prog Brain Res. 2007;163:43‐61. 17765711 10.1016/S0079-6123(07)63003-9

[17] Dolorfo CL , Amaral DG . Entorhinal cortex of the rat: topographic organization of the cells of origin of the perforant path projection to the dentate gyrus. J Comp Neurol. 1998;398:25‐48. 9703026

[18] Zhang SJ , Ye J , Couey JJ , Witter M , Moser EI , Moser MB . Functional connectivity of the entorhinal‐hippocampal space circuit. Philos Trans R Soc Lond B Biol Sci. 2014;369:20120516. 24366130 10.1098/rstb.2012.0516 PMC3866440

[19] Hyman BT , Van Hoesen GW , Kromer LJ , Damasio AR . Perforant pathway changes and the memory impairment of Alzheimer's disease. Ann Neurol. 1986;20:472‐481. 3789663 10.1002/ana.410200406

[20] Moryś J , Sadowski M , Barcikowska M , Maciejewska B , Narkiewicz O . The second layer neurones of the entorhinal cortex and the perforant path in physiological ageing and Alzheimer's disease. Acta Neurobiol Exp. 1994;54:47‐53. 8023713

[21] Augustinack JC , Helmer K , Huber KE , Kakunoori S , Zollei L , Fischl B . Direct visualization of the perforant pathway in the human brain with ex vivo diffusion tensor imaging. Front Hum Neurosci. 2010;4:42. 20577631 10.3389/fnhum.2010.00042 PMC2889718

[22] Yassa MA , Muftuler LT , Stark CE . Ultrahigh‐resolution microstructural diffusion tensor imaging reveals perforant path degradation in aged humans in vivo. Proc Natl Acad Sci U S A. 2010;107:12687‐12691. 20616040 10.1073/pnas.1002113107 PMC2906542

[23] Augustinack JC , van der Kouwe AJ , Blackwell ML , et al. Detection of entorhinal layer II using 7Tesla [corrected] magnetic resonance imaging. Ann Neurol. 2005;57:489‐494. 15786476 10.1002/ana.20426 PMC3857582

[24] Assaf Y . Imaging laminar structures in the gray matter with diffusion MRI. Neuroimage. 2019;197:677‐688. 29309898 10.1016/j.neuroimage.2017.12.096

[25] Uchida Y , Onda K , Hou Z , Troncoso JC , Mori S , Oishi K . Microstructural neurodegeneration of the entorhinal‐hippocampus pathway along the Alzheimer's disease continuum. J Alzheimers Dis. 2023;95:1107‐1117. 37638442 10.3233/JAD-230452 PMC10578220

[26] Hyman BT , Phelps CH , Beach TG , et al. National institute on aging‐Alzheimer's association guidelines for the neuropathologic assessment of Alzheimer's disease. Alzheimers Dement. 2012;8:1‐13. 22265587 10.1016/j.jalz.2011.10.007 PMC3266529

[27] Jack CR Jr , Andrews JS , Beach TG , et al. Revised criteria for diagnosis and staging of Alzheimer's disease: Alzheimer's association workgroup. Alzheimers Dement. 2024;20(8):5143‐5169. 38934362 10.1002/alz.13859 PMC11350039

[28] Attems J , Toledo JB , Walker L , et al. Neuropathological consensus criteria for the evaluation of Lewy pathology in post‐mortem brains: a multi‐centre study. Acta Neuropathol. 2021;141:159‐172. 33399945 10.1007/s00401-020-02255-2 PMC7847437

[29] Nelson PT , Dickson DW , Trojanowski JQ , et al. Limbic‐predominant age‐related TDP‐43 encephalopathy (LATE): consensus working group report. Brain. 2019;142:1503‐1527. 31039256 10.1093/brain/awz099 PMC6536849

[30] Aggarwal M , Mori S , Shimogori T , Blackshaw S , Zhang J . Three‐dimensional diffusion tensor microimaging for anatomical characterization of the mouse brain. Magn Reson Med. 2010;64:249‐261. 20577980 10.1002/mrm.22426 PMC2915547

[31] Jiang H , van Zijl PC , Kim J , Pearlson GD , Mori S . DtiStudio: resource program for diffusion tensor computation and fiber bundle tracking. Comput Methods Programs Biomed. 2006;81:106‐116. 16413083 10.1016/j.cmpb.2005.08.004

[32] Haynor DR , Li Y , Jiang H , Mori S , Ourselin S , (2012) Medical Imaging 2012: Image Processing.

[33] Li Y , Shea SM , Lorenz CH , Jiang H , Chou MC , Mori S . Image corruption detection in diffusion tensor imaging for post‐processing and real‐time monitoring. PLoS One. 2013;8:e49764. 24204551 10.1371/journal.pone.0049764 PMC3808367

[34] Kellner E , Dhital B , Kiselev VG , Reisert M . Gibbs‐ringing artifact removal based on local subvoxel‐shifts. Magn Reson Med. 2016;76:1574‐1581. 26745823 10.1002/mrm.26054

[35] Tournier JD , Smith R , Raffelt D , et al. MRtrix3: a fast, flexible and open software framework for medical image processing and visualisation. Neuroimage. 2019;202:116137. 31473352 10.1016/j.neuroimage.2019.116137

[36] Mori S , Crain BJ , Chacko VP , van Zijl PC . Three‐dimensional tracking of axonal projections in the brain by magnetic resonance imaging. Ann Neurol. 1999;45:265‐269. 9989633 10.1002/1531-8249(199902)45:2<265::aid-ana21>3.0.co;2-3

[37] Mori S , Kageyama Y , Hou Z , et al. Elucidation of white matter tracts of the human amygdala by detailed comparison between high‐resolution postmortem magnetic resonance imaging and histology. Front Neuroanat. 2017;11:16. 28352217 10.3389/fnana.2017.00016 PMC5348491

[38] Oishi K , Mori S , Troncoso JC , Lenz FA . Mapping tracts in the human subthalamic area by 11.7T ex vivo diffusion tensor imaging. Brain Struct Funct. 2020;225:1293‐1312. 32303844 10.1007/s00429-020-02066-x PMC7584118

[39] Pettigrew C , Soldan A , Zhu Y , et al. Cortical thickness in relation to clinical symptom onset in preclinical AD. Neuroimage Clin. 2016;12:116‐122. 27408796 10.1016/j.nicl.2016.06.010 PMC4932610

[40] Pettigrew C , Soldan A , Zhu Y , et al. Cognitive reserve and cortical thickness in preclinical Alzheimer's disease. Brain Imaging Behav. 2017;11:357‐367. 27544202 10.1007/s11682-016-9581-y PMC5743433

[41] Liu Y , Paajanen T , Zhang Y , et al. Analysis of regional MRI volumes and thicknesses as predictors of conversion from mild cognitive impairment to Alzheimer's disease. Neurobiol Aging. 2010;31:1375‐1385. 20447732 10.1016/j.neurobiolaging.2010.01.022

[42] Rose SE , Janke AL , Chalk JB . Gray and white matter changes in Alzheimer's disease: a diffusion tensor imaging study. J Magn Reson Imaging. 2008;27:20‐26. 18050329 10.1002/jmri.21231

[43] Gao J , Cheung RT , Lee TM , et al. Possible retrogenesis observed with fiber tracking: an anteroposterior pattern of white matter disintegrity in normal aging and Alzheimer's disease. J Alzheimers Dis. 2011;26:47‐58. 21558648 10.3233/JAD-2011-101788

[44] Zeineh MM , Palomero‐Gallagher N , Axer M , et al. Direct visualization and mapping of the spatial course of fiber tracts at microscopic resolution in the human hippocampus. Cereb Cortex. 2017;27:1779‐1794. 26874183 10.1093/cercor/bhw010 PMC5963820

[45] Vachha B , Huang SY . MRI with ultrahigh field strength and high‐performance gradients: challenges and opportunities for clinical neuroimaging at 7 T and beyond. Eur Radiol Exp. 2021;5:35. 34435246 10.1186/s41747-021-00216-2 PMC8387544

[46] Oh SH , Bilello M , Schindler M , Markowitz CE , Detre JA , Lee J . Direct visualization of short transverse relaxation time component (ViSTa). Neuroimage. 2013;83:485‐492. 23796545 10.1016/j.neuroimage.2013.06.047 PMC3815972

[47] Walhovd KB , Johansen‐Berg H , Káradóttir RT . Unraveling the secrets of white matter—bridging the gap between cellular, animal and human imaging studies. Neuroscience. 2014;276:2‐13. 25003711 10.1016/j.neuroscience.2014.06.058 PMC4155933

[48] Tillema JM , Pirko I . Neuroradiological evaluation of demyelinating disease. Ther Adv Neurol Disord. 2013;6:249‐268. 23858328 10.1177/1756285613478870 PMC3707351

[49] Granger SJ , Colon‐Perez L , Larson MS , et al. Reduced structural connectivity of the medial temporal lobe including the perforant path is associated with aging and verbal memory impairment. Neurobiol Aging. 2023;121:119‐128. 36434930 10.1016/j.neurobiolaging.2022.10.012 PMC10249650

[50] Mori S , Zhang J . Principles of diffusion tensor imaging and its applications to basic neuroscience research. Neuron. 2006;51:527‐539. 16950152 10.1016/j.neuron.2006.08.012

[51] Das SR , Ilesanmi A , Wolk DA , Gee JC . Beyond macrostructure: is there a role for radiomics analysis in neuroimaging? Magn Reson Med Sci. 2024;23:367‐376. 38880615 10.2463/mrms.rev.2024-0053 PMC11234947

[52] Kalus P , Slotboom J , Gallinat J , et al. Examining the gateway to the limbic system with diffusion tensor imaging: the perforant pathway in dementia. Neuroimage. 2006;30:713‐720. 16337815 10.1016/j.neuroimage.2005.10.035

[53] Widjaja E , Wei X , Vidarsson L , Moineddin R , Macgowan CK , Nilsson D . Alteration of diffusion tensor parameters in postmortem brain. Magn Reson Imaging. 2009;27:865‐870. 19152773 10.1016/j.mri.2008.11.009

[54] Shatil AS , Uddin MN , Matsuda KM , Figley CR . Quantitative ex vivo MRI changes due to progressive formalin fixation in whole human brain specimens: longitudinal characterization of diffusion, relaxometry, and myelin water fraction measurements at 3T. Front Med. 2018;5:31. 10.3389/fmed.2018.00031 PMC5826187 29515998

[55] Babulal GM , Quiroz YT , Albensi BC , et al. Perspectives on ethnic and racial disparities in Alzheimer's disease and related dementias: update and areas of immediate need. Alzheimer's & Dementia. 2019;15:292‐312. 10.1016/j.jalz.2018.09.009 PMC6368893 30555031

