# Supplementary material for: Quantification of perforant path fibers for early detection of Alzheimer's disease
Source: Alzheimers Dement. 2025 Apr 6;21(4):e70142. doi: 10.1002/alz.70142 (PMC11972979; doi:10.1002/alz.70142)
Supplement: Supplementary file 1 — Supporting Information [file ALZ-21-e70142-s003.pdf]

# Supplementary Materials

**Supplementary Table 1.** ABC score of Alzheimer's disease neuropathological changes

**Supplementary Table 2.** Differences in age, sex, and brain weight among ABC scores of Alzheimer's disease neuropathological changes

**Supplementary Table 3.** Differences in age, sex, and brain weight among clinical diagnoses of Alzheimer's disease

**Supplementary Table 4.** Anatomical MRI parameters across the groups of clinical AD diagnoses with the exclusion of a non-AD case with LATE

**Supplementary Table 5.** Sensitivity analysis for the perforant path (PP) fiber counts and lengths with varying tractography parameters

**Supplementary Figure 1.** Graphical methods for measuring the perforant path (PP) ratio

**Supplementary Figure 2.** Graphical methods for measuring the thickness of the entorhinal cortex (ERC)

**Supplementary Video 1.** The perforant path fibers were reconstructed using the DtiStudio software with the fiber assignment by continuous tracking deterministic algorithm

The supplemental materials have been provided by the authors to give readers additional information about their work.

**Supplementary Table 1.** ABC score of Alzheimer's disease neuropathological changes

| Score | A (Thal amyloid phase) | B (Braak NFT stage) | C (CERAD) |
|---|---|---|---|
| 0 | 0 | None | None |
| 1 | 1 or 2 | I or II | Sparse |
| 2 | 3 | III or IV | Moderate |
| 3 | 4 or 5 | V or VI | Frequent |

**Supplementary Table 2.** Differences in age, sex, and brain weight among ABC scores of Alzheimer's disease neuropathological changes

| | **A0 (N=10)** | **A1 (N=4)** | **A2 (N=3)** | **A3 (N=3)** | ***P* value** |
|---|---|---|---|---|---|
| **Age, y (SD)** | 62.8 (13.7) | 76.4 (14.5) | 71.5 (4.9) | 87.7 (4.0) | 0.13 |
| **Female, N (%)** | 3 (30) | 2 (40) | 2 (100) | 1 (33) | 0.32 |
| **Brain weight, g (SD)** | 1394 (170) | 1254 (115) | 1330 (170) | 1290 (110) | 0.39 |

| | **B0 (N=3)** | **B1 (N=6)** | **B2 (N=8)** | **B3 (N=3)** | ***P* value** |
|---|---|---|---|---|---|
| **Age, y (SD)** | 54.0 (4.6) | 65.8 (16.1) | 76.2 (11.8) | 86.5 (4.9) | 0.09 |
| **Female, N (%)** | 1 (33) | 2 (33) | 4 (144) | 1 (50) | 0.96 |
| **Brain weight, g (SD)** | 1477 (133) | 1347 (195) | 1294 (118) | 1290 (155) | 0.35 |

| | **C0 (N=10)** | **C1 (N=3)** | **C2 (N=4)** | **C3 (N=3)** | ***P* value** |
|---|---|---|---|---|---|
| **Age, y (SD)** | 62.8 (13.7) | 84.0 (8.5) | 71.5 (12.2) | 87.7 (4.0) | 0.07 |
| **Female, N (%)** | 3 (30) | 2 (100) | 2 (40) | 1 (33) | 0.32 |
| **Brain weight, g (SD)** | 1394 (170) | 1170 (71) | 1294 (104) | 1330 (130) | 0.25 |

The *P* values for age and brain weight were calculated using an analysis of variance, while the *P* value for sex was determined using the Chi-square test.

**Supplementary Table 3.** Differences in age, sex, and brain weight among clinical diagnoses of Alzheimer's disease

| | **Non-AD (N=10)** | **Preclinical AD (N=6)** | **AD dementia (N=4)** | ***P* value** |
|---|---|---|---|---|
| **Age, y (SD)** | 62.8 (13.7) | 75.0 (13.4) | 84.5 (7.1) | 0.07 |
| **Female, N (%)** | 3 (30) | 3 (50) | 2 (50) | 0.66 |
| **Brain weight, g (SD)** | 1394 (170) | 1247 (105) | 1330 (120) | 0.18 |

The *P* values for age and brain weight were calculated using an analysis of variance, while the *P* value for sex was determined using the Chi-square test.

**Supplementary Table 4.** Anatomical MRI parameters across the groups of clinical AD diagnoses with the exclusion of a non-AD case with LATE. The perforant path (PP) ratio, mean fractional anisotropy (FA) value of the presubiculum (PreS), number and length of PP fibers, and entorhinal cortex (ERC) thickness were compared across the groups along the AD continuum (non-AD *vs.* preclinical AD *vs.* AD dementia).

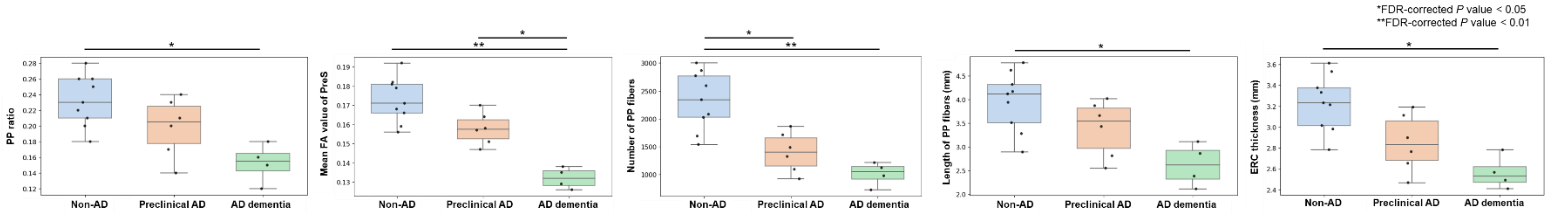

**Supplementary Table 5.** Sensitivity analysis for the perforant path (PP) fiber counts and lengths with varying tractography parameters

| **FA threshold** | **0.05** | **0.10 (baseline)** | **0.15** |
|---|---|---|---|
| PP fiber counts | 1724 ± 886 | 1667 ± 835 | 1585 ± 790 |
| PP fiber lengths, mm | 3.31 ± 1.10 | 3.48 ± 1.06 | 3.55 ± 1.02 |

| **Angle threshold** | **50°** | **60° (baseline)** | **70°** |
|---|---|---|---|
| PP fiber counts | 1691 ± 855 | 1667 ± 835 | 1633 ± 812 |
| PP fiber lengths, mm | 3.40 ± 1.12 | 3.48 ± 1.06 | 3.51 ± 1.04 |

| **Minimum fiber length** | **4 pixels** | **5 pixels (baseline)** | **6 pixels** |
|---|---|---|---|
| PP fiber counts | 1770 ± 902 | 1667 ± 835 | 1533 ± 765 |
| PP fiber lengths, mm | 3.27 ± 1.16 | 3.48 ± 1.06 | 3.62 ± 1.09 |

Data are means ± standard deviation.

Abbreviation: FA, fractional anisotropy.

**Supplementary Figure 1.** Graphical methods for measuring the perforant path (PP) ratio. PP fibers on the presubiculum (PreS) were identified as dark striate intensities on 3DT2WI. **(A)** The red bounding boxes are 5× magnified to clearly visualize PP fibers on PreS. **(B)** Using the RoiEditor software, the numbers of voxels on PP fibers (cyan-bordered areas) and PreS (red-colored areas) were counted across selected slices of 2D coronal planes in the left medial temporal lobe. The coronal slices were selected based on the following criteria: from the first slice where the dentate gyrus of the hippocampus is initially observed to the last slice where the dentate gyrus of the hippocampus is split into the inner and outer portions. The ratio of voxel counts of PP fibers to those of PreS, named the PP ratio, was then calculated.

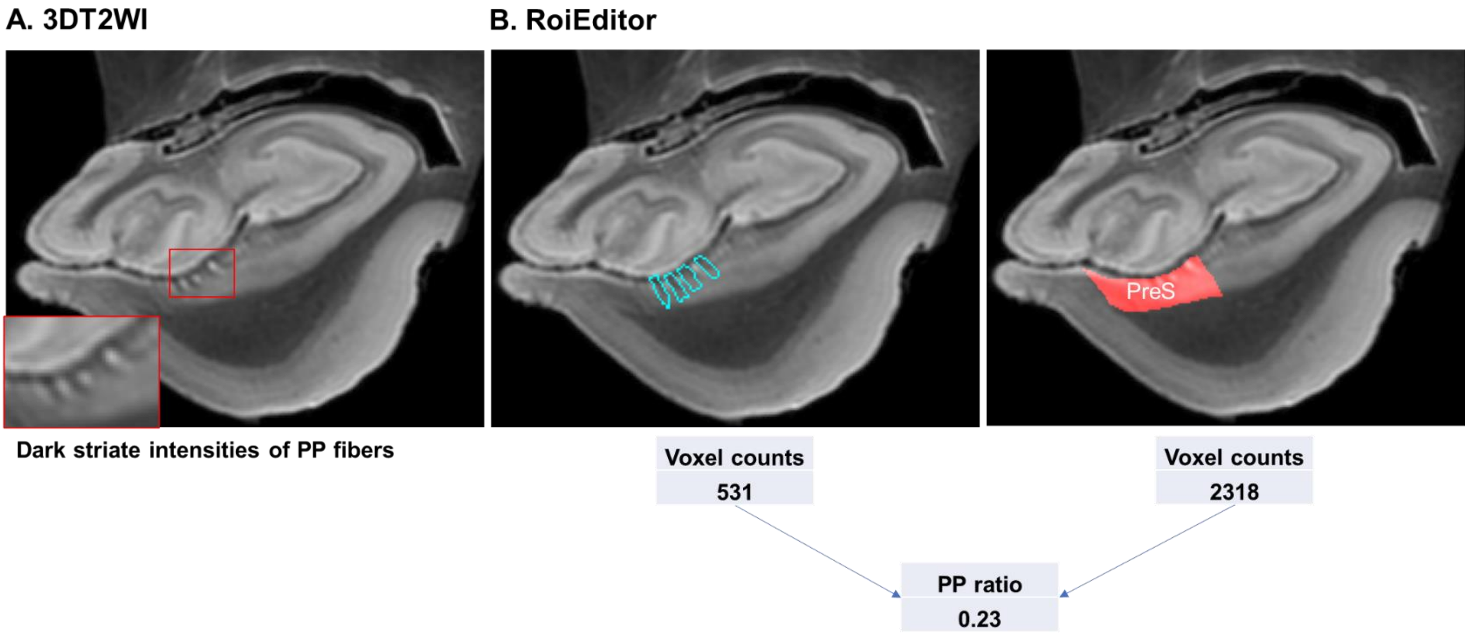

**Supplementary Figure 2.** Graphical methods for measuring the thickness of the entorhinal cortex (ERC). **(A)** In the representative gross finding of the brain specimen, the red lines indicate the medial (a), middle (b), and lateral (c) parts of ERC, respectively. **(B)** Using the ITK-SNAP software, each line was manually measured, and then the mean ERC thickness was calculated. DG: dentate gyrus; PreS: presubiculum; SUB: subiculum.

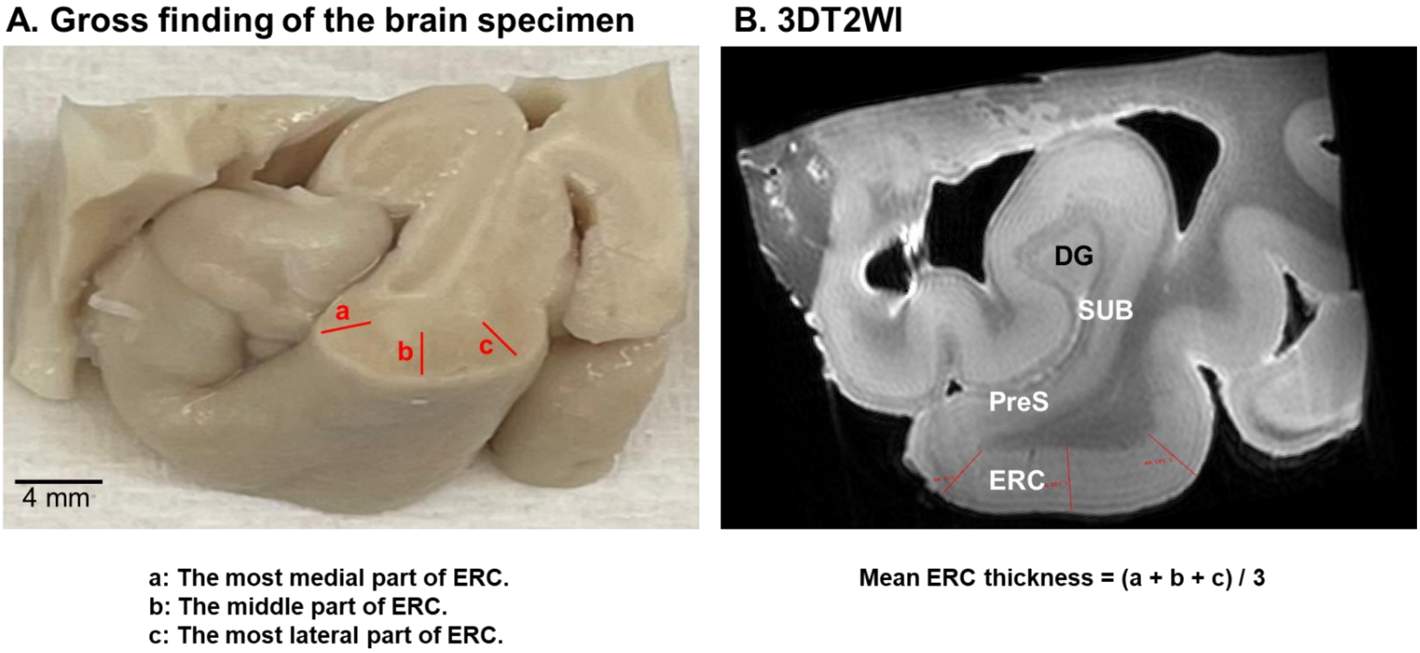

**Supplementary Video 1.** The perforant path fibers were reconstructed using the DtiStudio software with the fiber assignment by continuous tracking deterministic algorithm. The fibers are visualized as a tract extending from the entorhinal cortex to the dentate gyrus of the hippocampus, passing through the presubiculum.
